# Feasibility of a Digital Health Intervention to Improve Diet Quality Among Women With High Blood Pressure: Randomized Controlled Feasibility Trial

**DOI:** 10.2196/17536

**Published:** 2020-12-07

**Authors:** Dori M Steinberg, Melissa C Kay, Laura P Svetkey, Sandy Askew, Jacob Christy, Jasmine Burroughs, Hira Ahmed, Gary G Bennett

**Affiliations:** 1 Duke University School of Nursing Durham, NC United States; 2 Duke Global Digital Health Science Center Duke Global Health Institute Durham, NC United States; 3 Duke Center for Childhood Obesity Research Department of Pediatrics Duke University Medical Center Durham, NC United States; 4 Sarah W Stedman Nutrition and Metabolism Center Duke University Medical Center Durham, NC United States; 5 Division of Nephrology Department of Medicine Duke University Medical Center Durham, NC United States; 6 Department of Family Medicine and Community Health Duke University Durham, NC United States; 7 Department of Psychology and Neuroscience Duke University Durham, NC United States

**Keywords:** hypertension, DASH dietary pattern, digital health, nutrition, women’s health, mHealth

## Abstract

**Background:**

Over 100 million individuals have high blood pressure, and more than half of them are women. The Dietary Approaches to Stop Hypertension (DASH) dietary pattern is a proven lifestyle approach to lower blood pressure, yet population-level adherence is poor. Innovative strategies that promote DASH are needed.

**Objective:**

This paper aims to improve adherence to the DASH diet among women with hypertension or prehypertension.

**Methods:**

We conducted a 3-month randomized controlled feasibility trial comparing app-based diet tracking (active comparator) to app-based diet tracking plus feedback on DASH adherence via text message (intervention). The intervention platform extracted nutrient data from the app, compared it to DASH recommendations, and sent tailored feedback text messages. Outcomes included the number of days participants tracked their diet, changes in their DASH adherence score, and blood pressure.

**Results:**

The women (N=59) had a mean age of 49.9 (SD 11.9) years and were primarily non-Hispanic White (41/59, 69%) and college educated (49/59, 83%). The mean baseline DASH score was 2.3 (SD 1.3). At 3 months, the intervention and active comparator participants had similar mean days tracked per week (4.2, SD 2.1 days vs 4.6, SD 2.7 days; *P*=.54) and mean changes in their DASH score (0.8, 95% CI 0.2-1.5 vs 0.8, 95% CI 0.4-1.2; *P*=.75). Intervention participants had lower systolic (mean difference: –2.8 mmHg, 95% CI –1.8 to 7.4; *P*=.23) and diastolic (mean difference: –3.6 mmHg, 95% CI –0.2 to 7.3; *P*=.07) blood pressure compared with active comparator participants. Most intervention participants (23/29, 79%) said they would recommend the DASH Cloud intervention to a friend or family member. However, only 34% (10/59) indicated that the feedback text messages helped them reach their diet goals.

**Conclusions:**

A digital health intervention to improve DASH adherence is feasible and produces moderately high engagement among women with elevated blood pressure. The intervention did not enhance DASH adherence over diet tracking alone but resulted in greater reductions in blood pressure. Larger studies are needed to determine how digital health interventions can improve population-level adherence to DASH.

**Trial Registration:**

ClinicalTrials.gov NCT03215472; https://clinicaltrials.gov/ct2/show/study/NCT03215472

## Introduction

More than 100 million Americans have high blood pressure [1], the primary risk factor for heart disease and stroke, which are two of the leading causes of death in the United States [2]. In 2017, the American Heart Association implemented new guidelines for the detection, prevention, and treatment of high blood pressure [3]. These guidelines lowered the thresholds for what is considered an optimal blood pressure, which resulted in about 30 million more Americans requiring treatment for high blood pressure, including 13 million more women [1,3,4]. Many of these women, however, present with atypical risk factors for high blood pressure and may not receive the treatment they need [5]. Participation of women in blood pressure trials is disproportionately lower, and there is a need for a lifestyle treatment that is tailored for women [6]. Lifestyle treatment is indicated for all individuals with high blood pressure regardless of whether medication is also indicated, but it is the first line of treatment for those in the elevated blood pressure category [3].

The Dietary Approaches to Stop Hypertension (DASH) dietary pattern is an evidence-based lifestyle treatment to lower blood pressure [7]. DASH emphasizes nutrient-rich foods, such as fruits and vegetables, whole grains, and low-fat dairy products, and limits red meat and processed foods [8]. DASH was specifically designed to increase nutrient consumption of calcium, magnesium, potassium, fiber, and protein and reduce the intake of saturated fat, total fat, and cholesterol [8]. The initial DASH trial, which compared a typical US diet to the DASH dietary pattern among adults with nonmedicated high blood pressure, resulted in a decrease of systolic and diastolic blood pressure by 5.5 and 3.0 mmHg, respectively [7]. The blood pressure–lowering effects of DASH have been replicated in multiple trials [9-11].

As such, DASH is recommended in national blood pressure [3,12] and dietary [13] guidelines. However, the adoption of DASH on a population level remains poor [14]. Fewer than 1% of US adults are fully adherent to DASH, and only 20% meet half of the recommendations of DASH [9,15]. This indicates a need for improved dissemination and support to increase population uptake and adherence to DASH.

Smartphones are a promising channel for disseminating and supporting DASH adherence. More than three-quarters of Americans have smartphones, with high rates of ownership across all socioeconomic strata [16]. Diet and fitness smartphone apps account for 43% [16,17] of mobile medical apps, with the most popular apps having 50 million users [18]. These publicly available apps are primarily diet trackers that ask users to enter all foods and beverages consumed and then link those data with nutritional databases. Although popular, these apps lack evidence-based components known to produce behavior change, such as consistent self-monitoring, goal setting, problem-solving skills, feedback, and social support [19,20]. To address this, we can create programs that combine these publicly available apps with evidence-based behavior change principles to improve dissemination of DASH.

In the current study, we created the DASH Cloud intervention. Using data collected through a commercial diet-tracking smartphone app, DASH Cloud provides tailored feedback via text messages about adherence to DASH, accompanied by motivational messages designed to support behavior change. We tested the feasibility of the intervention among women with high blood pressure using a randomized controlled study design. The 3-month trial compared the DASH Cloud intervention to diet tracking alone on adherence to DASH. Using the principles of feasibility outlined by Leon et al [21], feasibility was determined based on successful recruitment and retention and the successful implementation of the intervention. We hypothesized that women receiving the 3-month DASH Cloud intervention would have (1) greater rates of diet tracking and (2) greater adherence to DASH.

## Methods

### Study Design and Eligibility

We recruited women aged 21 to 70 years with a BMI of >18.5 kg/m^2^. Women were eligible if they self-reported being diagnosed with hypertension, using medication for blood pressure, or having a recent systolic measurement of 120 to 159 mmHg or a diastolic blood pressure measurement of 80 to 99 mmHg. They were required to have a smartphone with the latest operating system or be willing to update to the current version, have a data plan, and be willing to receive daily text messages. Having an email address and fluency in English were also required. Participants were excluded if they had a cardiovascular event in the last 6 months or a current cancer diagnosis, had been institutionalized for a psychiatric disorder within the past year, or if they were pregnant, lactating, or enrolled in another dietary change study.

Eligible participants were redirected to an online consent form to review and sign, which included both a brief video that described the study and a written version. Participants then completed online surveys and attended baseline study visits at our study offices. During the first baseline visit, participants’ height, weight, blood pressure, and depression status were measured.

At the first baseline visit, participants downloaded and were instructed on how to use the Nutritionix diet-tracking app (Syndigo LLC) and asked to track their diet for a week to assess their ability to track using the study app. Participants came back for a second baseline visit 1 week later if they tracked a minimum of 4 consecutive days. During the second baseline visit, participants were randomized using a permuted block design with random block sizes ranging from 4 to 8. An allocation table was created by a biostatistician using Sealed Envelope and uploaded to a Research Electronic Data Capture (REDCap) project. Allocation was revealed to unblinded research staff upon random assignment by the REDCap software, allowing the research staff to orient the participant to either the DASH Cloud intervention or an active comparator arm. Participants had a follow-up visit after 3 months. Participants were compensated US $25 at the second baseline visit and the 3-month follow-up visit. All study protocols were in accordance with the ethical standards of Duke University and were approved by the Duke University Health Center institutional review board. The study was registered on ClinicalTrials.gov on July 12, 2017 (NCT03215472).

### Recruitment

Participants were recruited using a multipronged approach, including (1) flyers distributed to gyms, community centers, grocery stores, and health and wellness clinics throughout Raleigh, Durham, and Chapel Hill, North Carolina; (2) study details posted on Research Match, a national clinical trials registry that matches participants to studies; (3) social media posts on Twitter, Facebook, and Nextdoor; and (4) contact with participants who were ineligible for other studies but showed interest in similar behavioral studies.

### Intervention Description

The intervention included (1) daily diet tracking using the Nutritionix app; (2) daily or weekly text messages that included tailored feedback on adherence to DASH, motivational messages to boost adherence, and tips for specific dietary changes; (3) animated videos designed to increase skills around different topics related to DASH; and (4) the DASH booklet available from the National Heart, Lung, and Blood Institute (NHLBI) [22].

#### Daily Diet Tracking

All participants were asked to use Nutritionix daily and enter all foods and beverages consumed. Like many commercial diet apps, Nutritionix primarily provides tracking capabilities. The app interface includes options to enter foods by meal and time of day and includes small pictures of each food. Nutritionix also has a comprehensive and easily accessible nutrient database supporting entries into the app. This database includes all data available from the United States Department of Agriculture (USDA) Food Composition Database, data from restaurant chains, and foods added by registered dietitians staffed by Nutritionix. Dietary data were automatically uploaded from the Nutritionix app daily via an application programing interface (API). The API connects the Nutritionix server to the DASH Cloud intervention delivery system, which uses servers stored in a platform operated out of the Duke Global Digital Health Science Center called Prompt. As is common with nutrient databases [23], not all foods available in the Nutritionix database had complete nutrient data. For example, magnesium and potassium were nutrients we used in our DASH algorithm that were often not available for many foods. As such, to ensure we had complete nutrient data, our registered dietitians on staff manually filled in any missing nutrient data that were received via the Nutritionix API.

#### Feedback Texts

**Figure 1:**
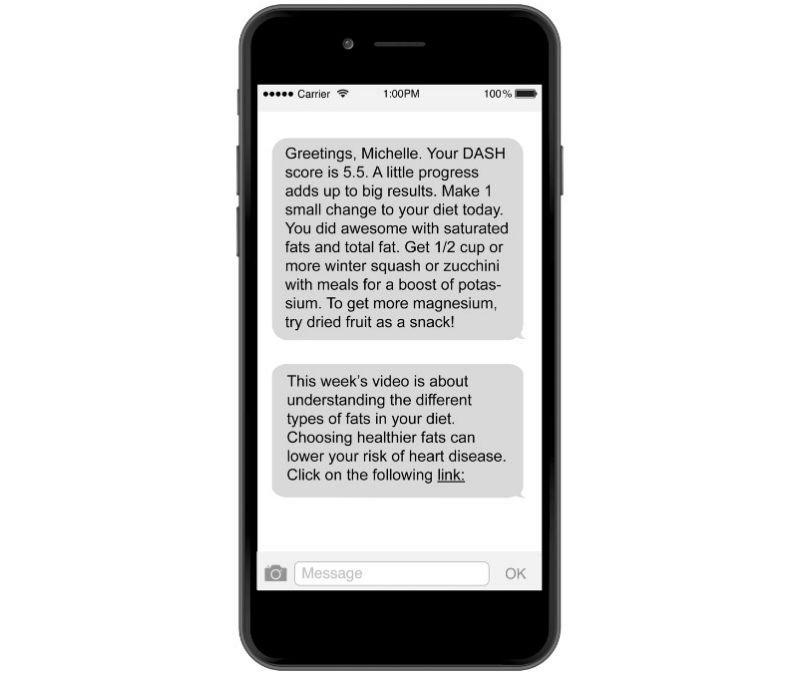
Sample text message sent to participants in the DASH Cloud intervention. DASH: Dietary Approaches to Stop Hypertension.

Using an automated algorithm, the intervention platform sent daily feedback via text message at 12:00 PM EST for the first 2 weeks and weekly feedback via text message for the remainder of the study (Figure 1). We opted to send the texts daily during the first 2 weeks to provide feedback proximal to the specific food choice. After 2 weeks, we switched to giving feedback weekly to reduce the potential burden from texting daily. This decision was based on balancing the concept of proximal feedback with the perceived burden from frequent notifications. The feedback message began with a personalized greeting (eg, “Hello, [name]”) and the participant’s DASH diet adherence score from the previous day (eg, “Your DASH score yesterday was 5.5”). This score was calculated using the Mellen et al [15] index to evaluate DASH adherence based on nutrient targets. The score uses a 9-point scale based on the previous day’s intake for potassium, sodium, magnesium, calcium, saturated fat, total fat, total protein, cholesterol, and fiber. Our algorithm compared the total intake and the recommended targets for these nutrients in DASH. Similar to the Mellen et al [15] index, our software platform was coded to automatically apply a score of 0, 0.5, or 1 to each nutrient based on its difference from the recommended target. The texts included feedback on which nutrients met the target and tips for improving nutrient intake for those that were suboptimal (eg, “You did best with reducing saturated fat and boosting your fiber intake and seemed to struggle with getting enough potassium and magnesium”).

Feedback also included motivational messages regarding changes in the overall DASH score and prompted participants to reflect on which dietary choices most impacted their DASH score. Each week on Sunday afternoons, we also sent a separate text message about a different topic related to DASH (eg, “Dining out on DASH,” with links to animated educational videos). Overall, these text messages were intended to be motivational by offering behavioral tips to reinforce dietary change and providing social support for women at risk for hypertension and cardiovascular disease. We aimed to tailor the intervention content for women and, in particular, improve understanding of the risks specific to women regarding cardiovascular disease. Participants were not expected to respond, and an automatic reply was sent if participants did respond indicating that they could reach out for support to our staff via email.

### Active Comparator Arm

Participants in the active comparator arm were asked to track their diet daily using the Nutritionix app and were shown a video that introduced the DASH dietary pattern. They were asked to try to follow DASH guidelines. However, unlike the intervention arm, they did not receive any of the text messages, which included tailored feedback and skills training videos. Active comparator participants received the same publicly available booklet from NHLBI with information about the DASH diet.

### Measures

#### Baseline Sociodemographic and Psychosocial Variables

To characterize participants, we measured various sociodemographic and psychosocial variables. Sociodemographic measures were collected at baseline using standard survey questions used in previous studies. This included age, race and ethnicity, educational attainment, marital status, employment, insurance status, and the number of children within the household. Depression was assessed using the validated 8-item survey from the Patient Health Questionnaire [24]. The scale ranges from 0 to 24, and a score of >10 indicates depression.

#### Anthropometrics

Weight and height were collected by study staff and BMI was calculated for each participant. Height was measured twice and recorded to the nearest 0.1 cm using a wall-mounted height rod (Portrod; Health O Meter). Weight was measured twice and recorded to the nearest 0.1 kg using a scale (Scale-Tronix 5005; Welch Allyn).

#### Engagement With Diet Tracking

Engagement was operationalized as a valid day tracked, determined by looking at overall caloric intake. A day with missing data or a daily caloric level of <600 calories was considered to be a day that did not have complete tracking data and was marked invalid [25]. We assessed the average days tracked per week and calculated the proportion of participants who tracked at least 5 days per week.

#### DASH Diet Adherence

To assess change in DASH adherence, dietary intake was measured using the Automated Self-Administered 24-hour (ASA24) recall tool from the National Cancer Institute. The ASA24 is an automated tool that uses the USDA’s validated multiple-pass method to elicit intake throughout a given day [26]. Using an unannounced protocol, participants were asked via email to complete 1 weekend day and 1 weekday of dietary intake within a 2-week period. The ASA24 provides comprehensive nutrient data on all foods and beverages consumed during the previous 24-hour period. These data were used to calculate a nutrient-based DASH index score, developed by Mellen and colleagues [15], as mentioned above. Nutrient targets can be found in the “Change in DASH Adherence Across Arms” section and were standardized to total calorie intake. Individual nutrient scores were summed to calculate a total DASH adherence score. As such, the score range is 0 to 9, with higher scores indicative of greater adherence and a score of 9 indicating full adherence to the DASH dietary pattern.

#### Blood Pressure

Participants confirmed that they had not consumed coffee or used tobacco 30 minutes before the blood pressure measurement. We standardized our procedures to use the right arm unless a participant indicated that it was medically contraindicated, and we measured arm circumference to determine blood pressure cuff size. After resting for 5 minutes, blood pressure was measured 3 times with 30-second intervals in between each measurement using a blood pressure monitor (HEM-907XL; Omron Healthcare). Using the average blood pressure measurement, we categorized participants based on their severity of hypertension [3].

#### Intervention Satisfaction

At 3 months, using surveys adapted from our previous studies, we assessed perceived ease and usefulness of the Nutritionix app across both study arms and overall satisfaction with the DASH Cloud intervention within the intervention arm only. Using a 5-point Likert scale from strongly disagree to strongly agree, we asked participants about their perceptions of all elements of the intervention, including the ease and usefulness of the Nutritionix app, the DASH score in the feedback, the personalization of the texts, the timing of the texts, and whether an individual would recommend this program to others. The “Satisfaction With Intervention Activities” section describes the intervention satisfaction questions we asked the intervention arm only and the proportion that responded in agreement.

### Analysis

For descriptive analyses, normally distributed variables were summarized and reported as means and standard deviations. Engagement was analyzed using a 2-tailed *t* test to compare overall mean engagement over the course of the study. Engagement trends over time were analyzed using a random-intercept mixed model with unstructured covariance. The model included a continuous variable for time in weeks, a treatment group variable, and a time-by-group interaction term. To assess changes in the DASH adherence score, we conducted a primary analysis that treated invalid baseline and 3-month data as missing. Using standard protocols for obtaining valid dietary intake [23], we defined invalid assessments as those having a mean daily caloric intake of <600 or >3500 calories or those that did not include at least 2 recalls from 1 weekend day and 1 weekday or that included recalls that were collected more than 2 weeks apart. To examine the effect of the treatment group over time on DASH adherence, controlling for baseline levels of DASH adherence, we conducted linear regression models assessing 3-month DASH scores and DASH component values by study arm and including baseline DASH adherence as a covariate. Within-group changes in DASH scores and components were estimated using repeated-measures analysis of variance methods. Sensitivity analysis models were conducted using the same methods while including measurements previously excluded as invalid in the per-protocol models. Between-group differences in 3-month systolic and diastolic blood pressure were also assessed using linear regression models and adjusting for baseline values. Missing 3-month values in the DASH and blood pressure models were addressed using intent-to-treat principles with maximum likelihood estimations. Analyses were conducted using SAS 9.4 (SAS Institute) software and *P* values with an α <.05 were considered statistically significant.

## Results

### Recruitment and Retention Rates

**Figure 2:**
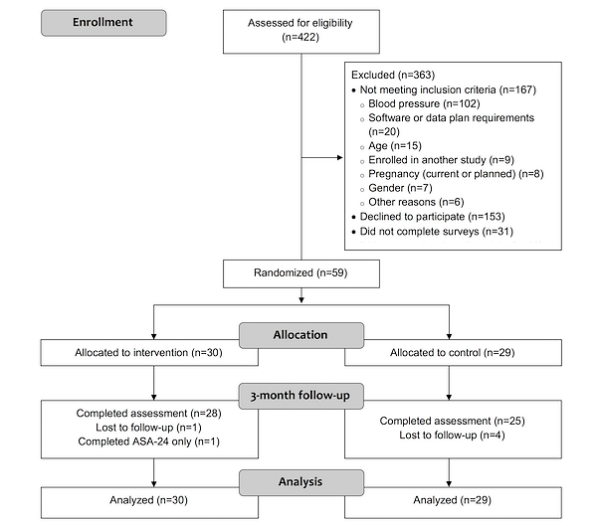
Study flow and CONSORT diagram. CONSORT: Consolidated Standards of Reporting Trials. ASA24: Automated Self-Administered 24-hour recall tool.

The recruitment period lasted from July 21, 2017, to November 5, 2017. The CONSORT (Consolidated Standards of Reporting Trials) diagram (Figure 2) shows the study flow for both recruitment and retention; 422 individuals filled out the screening on the DASH Cloud website, and 363 of those individuals were ineligible, declined participation, or did not complete baseline activities. The primary reason for ineligibility was not meeting the blood pressure criteria. Of the remaining eligible participants, 59 were then randomized to receive the DASH Cloud intervention (n=30) or the active comparator arm (n=29). At the end of the study, 90% (28/30 intervention, 25/29 control) of participants attended the final 3-month visit. The ASA24 surveys were collected outside the in-person 3-month visit and had different retention rates. Of the 59 total participants, 53 (90%; 29/30 intervention, 24/29 control) completed the ASA24 per protocol at baseline, and 46 (78%; 25/30 intervention, 21/29 control) completed it at 3 months; 43 (73%; 24/30 intervention, 19/29 control) completed it per protocol at both time points. All participants completed at least 1 ASA24 at baseline, and 55 of the 59 (93%) participants completed at least 1 diet recall at 3 months.

### Baseline Characteristics

As is shown in Table 1, participants (N=59) were all women with a mean age of 49.9 (SD 11.9) years and a mean BMI of 33.9 (SD 7.6) kg/m^2^ (Table 1). Most participants were non-Hispanic White (41/59, 69%), insured (57/59, 97%), college educated (49/59, 83%), employed (42/59, 71%), and married (36/59, 61%). At baseline, the mean systolic blood pressure was 122.9 (SD 14.2) mmHg and the mean diastolic blood pressure was 80.2 (SD 8.8) mmHg. The majority of participants (41/59, 69%) were classified as having elevated blood pressure according to updated blood pressure guidelines from the American Heart Association and the American College of Cardiology, which is defined as any measurement of systolic blood pressure over 120 mmHg or a diastolic blood pressure measurement over 80 mmHg. These updated guidelines have lowered the threshold for elevated blood pressure, and lifestyle treatments, such as DASH, are a first line of treatment for those meeting these criteria. There were no differences in baseline characteristics by study arm.

**Table 1:** Sociodemographic and clinical baseline characteristics of participants enrolled in the DASH Cloud intervention trial (N=59).

Characteristic		Total
Age (years), mean (SD)		49.9 (11.9)
BMI (kg/m^2^), mean (SD)		33.8 (7.6)
Systolic blood pressure (mmHg), mean (SD)		122.9 (14.2)
Diastolic blood pressure (mmHg), mean (SD)		80.2 (8.8)
Normal blood pressure category (defined as <120/80 mmHg), n (%)^a^		18 (31)
Self-reported use of blood pressure medications, n (%)		29 (49)
Married or living with partner, n (%)		36 (61)
Children in household, n (%)		23 (41)
Currently employed, n (%)		42 (71)
Currently insured, n (%)		57 (97)
**Education, n (%)**		
	Vocational or trade school or some college after high school	5 (8)
	Associate degree from college or university	5 (8)
	College degree	15 (25)
	Postgraduate degree from college or university	34 (58)
**Race/ethnicity, n (%)**		
	Non-Hispanic White	41 (69)
	Non-Hispanic Black	10 (17)
	Hispanic Black	2 (3)
	Hispanic White	2 (3)
	Other (Asian, multiracial, or unreported)	4 (7)
**Depression, n (%)^b^**		
	Minimal depression (score of 0-4)	37 (63)
	Mild depression (score of 5-9)	18 (31)
	Moderate depression (score of 10-14)	4 (6)

^a^Using the blood pressure treatment categories from the 2017 American Heart Association and the American College of Cardiology guidelines.

^b^Using the 8-item Patient Health Questionnaire scale, with scores ranging from 0 to 24.

### Engagement With Diet Tracking

**Figure 3:**
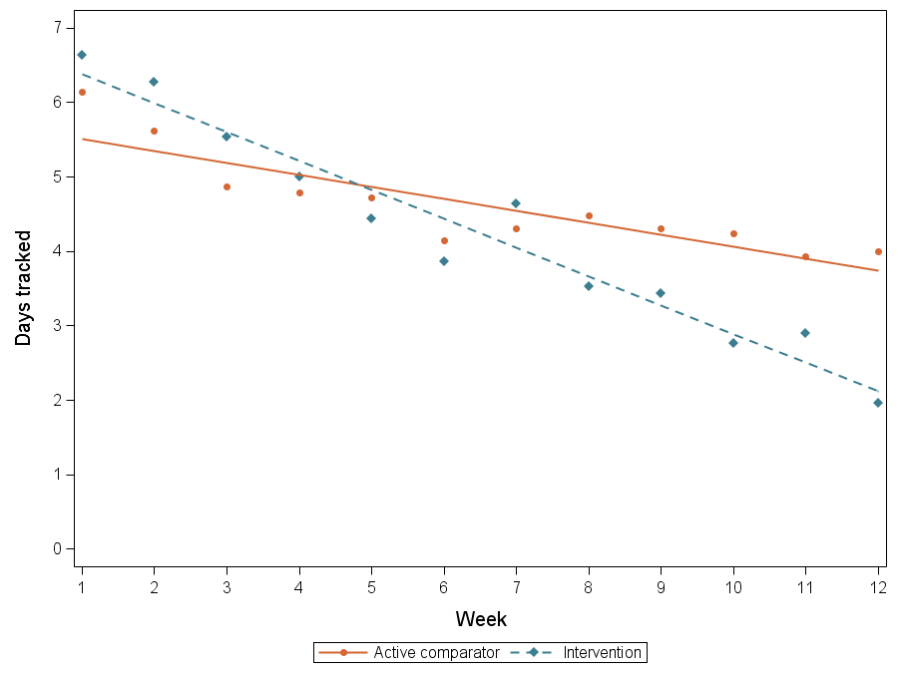
Predicted mean days of diet tracking over time by study arm.

Figure 3 shows the actual and predicted days tracked per week across study arms. The mean days tracked per week overall was slightly higher, although not statistically significant, in the active comparator arm compared with the intervention arm (mean 4.6, SD 2.7 days vs 4.2, SD 2.1 days; *P*=.54). Likewise, a higher proportion of active comparator participants tracked 5 days or more per week, on average, compared with intervention participants (18/29, 63% vs 14/30, 47%; *P*=.24). However, the intervention participants experienced a steeper reduction in diet-tracking engagement over time, with rates decreasing by 0.23 (95% CI 0.16-0.29) days per week (*P*<.001), which is about a day per month faster than the active comparator group.

### Change in DASH Adherence Across Arms

As is shown in Table 2, the mean DASH score at baseline was 2.2 (SD 1.3), and there were no significant differences across study arms (2.2, SD 1.3 in intervention vs 2.4, SD 1.3 in active comparator; *P*=.85). After we adjusted for baseline DASH scores, participants in the intervention arm had DASH scores that were 0.01 (95% CI –0.7 to 0.7) points lower than the active comparator arm at 3 months (*P*=.97). Both groups experienced significant increases in DASH scores at 3 months (intervention mean change: 0.8, 95% CI 0.2-1.5; *P*=.02; active comparator mean change: 0.8, 95% CI 0.4-1.2; *P*<.001). No significant differences were observed between groups on any nutrient component within the DASH score after adjusting for baseline between-group differences. Both groups experienced significant or marginally significant increases in fiber intake over the 3 months. Both groups also reported significant decreases in saturated fat. Within the intervention group, there was a significant increase in magnesium intake and a significant decrease in total fat consumption. Within the active comparator group, there was a significant decrease in total fat consumption and an increase in calcium.

Sensitivity analysis that included ASA24 data that were not valid per protocol produced results that were generally consistent with the per-protocol models using valid data. Data from all 59 participants were included, with 93% (55/59) of participants contributing 3-month data to these models.

**Table 2:** Change in DASH adherence score and DASH score components within and across study arms among participants in the DASH Cloud intervention trial (N=59).^a^

Nutrient	Standards	Intervention					Active comparator					Between-group difference	
		Baseline, mean (SD) (n=29)	3 months, mean (SD) (n=25)	Estimated mean difference (95% CI) (n=30)	*P* value	Baseline, mean (SD) (n=24)		3 months, mean (SD) (n=21)	Estimated mean difference (95% CI) (n=29)	*P* value	Adjusted mean difference (95% CI) (N=59)		*P* value
DASH^b^ score	0-9	2.2 (1.3)	3.1 (1.4)	0.8 (0.2 to 1.5)	.02	2.3 (1.3)		3.1 (1.3)	0.8 (0.4 to 1.2)	.001	–0.01 (–0.7 to 0.7)		.97
Total fat (% of total calories)	<27% of total daily kcal	37.9 (7.4)	37.6 (7.2)	–0.2 (–2.7 to 2.4)	.89	39.4 (7.1)		34.9 (6.8)	–3.9 (–6.7 to –1.0)	.01	3.1 (–0.2 to 6.3)		.06
Saturated fat (% total calories)	<6% of total daily kcal	12.6 (3.5)	11.4 (2.9)	–1.4 (–2.6 to –0.2)	.03	12.9 (2.8)		11.2 (3.0)	–1.5 (–2.9 to –0.1)	.03	0.1 (–1.4 to 1.5)		.90
Protein (% total calories)	>18% of total daily kcal	16.0 (3.9)	17.4 (5.2)	1.2 (–1.1 to 3.4)	.30	16.5 (4.1)		16.4 (3.1)	–0.01 (–1.7 to 1.7)	.99	1.0 (–1.3 to 3.4)		.39
Cholesterol (mg/1000 kcal)	<71.4 mg/1000 kcal per day	172.2 (84.8)	160.9 (101.1)	–13.2 (–61.9 to 35.4)	.58	167.1 (80.8)		133.2 (67.4)	–31.5 (–71.5 to 8.4)	.12	23.8 (–25.2 to 72.8)		.34
Fiber (g/1000 kcal)	>14.8 g/1000 kcal per day	9.1 (4.9)	11.0 (4.3)	2.1 (0.7 to 3.5)	.004	9.5 (3.7)		11.6 (4.8)	1.9 (–0.04 to 3.8)	.054	0.1 (–2.0 to 2.1)		.96
Magnesium (mg/1000 kcal)	>238 mg/1000 kcal per day	142.2 (32.0)	171.3 (45.7)	28.3 (7.5 to 49.0)	.01	157.3 (51.1)		173.6 (44.1)	14.0 (–6.9 to 34.9)	.18	5.7 (–19.1 to 30.5)		.65
Calcium (mg/1000 kcal)	>590 mg/1000 kcal per day	494.4 (170.9)	489.5 (222.9)	–12.5 (–96.9 to 71.9)	.76	433.6 (137.2)		544.3 (238.3)	112.3 (3.6 to 221.1)	.04	–96.5 (–221.1 to 28.1)		.13
Potassium (mg/1000 kcal)	>2238 mg/1000 kcal per day	1318.0 (420.3)	1489.8 (439.8)	168.7 (–23.4 to 360.9)	.08	1388 (323.0)		1559.2 (446.0)	163.8 (–33.4 to 360.9)	.10	–25.0 (–252.7 to 202.7)		.83
Sodium (mg)	<2400 mg per day	3223.6 (912.1)	3078.4 (975.1)	–187.3 (–602.8 to 228.1)	.36	2775.6 (860.4)		3024.1 (737.1)	241.4 (–205.5 to 688.4)	.28	–101.5 (–576.5 to 373.4)		.68

^a^Participants with an invalid ASA24 were treated as missing.

^b^DASH: Dietary Approaches to Stop Hypertension.

### Change in Blood Pressure Across Arms

After adjusting for baseline, participants in the intervention arm had systolic blood pressures that were, on average, 2.8 (95% CI –1.8 to 7.4) mmHg lower than the active comparator arm at 3 months, though not significantly (*P*=.23). Participants’ 3-month average diastolic blood pressure was lower in the intervention arm, on average, by 3.6 (95% CI –0.2 to 7.3) mmHg compared with the active comparator arm after adjusting for baseline, though not significantly (*P*=.07).

### Association Between Change in DASH Adherence and Change in Blood Pressure

Overall, changes in DASH scores and changes in blood pressure were inversely correlated. A 1-unit improvement in the DASH score was associated with a 2.5 (95% CI 0.5 to 4.5) mmHg decrease in systolic blood pressure (*r*=–0.34; *P*=.02) and a 1.6 (95% CI 0.05 to 3.3) mmHg decrease in diastolic blood pressure (*r*=–0.27; *P*=.05). The observed correlation was consistent between groups (*P*=.63). On average, the intervention group decreased by 2.7 (95% CI 0.4 to 5.0) mmHg in systolic blood pressure (*r*=–0.44; *P*=.03) and 1.3 (95% CI –1.0 to 3.6) mmHg in diastolic blood pressure (*r*=–0.23; *P*=.26) for each unit improvement in the DASH score over 3 months. The association in the active comparator group was slightly weaker, with a 1.7 (95% CI –2.1 to 5.4) mmHg decrease in systolic blood pressure (*r*=–0.20; *P*=.37) and a 1.8 (95% CI –0.8 to 4.4) mmHg decrease in diastolic blood pressure (*r*=–0.29; *P*=.16) per unit of DASH score improvement.

### Satisfaction With Intervention Activities

Across study arms, 82% (24/29) of intervention participants indicated with agreement or strong agreement that the Nutritionix app was easy to use. Half of participants (14/29, 50%) said that they would use the app frequently, and only 15% (4/29) indicated that the app was cumbersome to use. Within the intervention arm, the majority of intervention participants (23/29, 79%) said they would recommend the DASH Cloud intervention to a friend or family member (Table 3). Many participants (23/29, 79%) felt the texts were sent at a convenient time, and many (22/29, 76%) preferred a consistent time to receive the texts. Most (23/29, 79%) participants felt the DASH dietary pattern tips were easy to understand. Notably, only about half (16/29, 55%) of participants felt the feedback and DASH score were helpful; 45% (13/29) felt that the DASH score was motivating, and 41% (12/29) indicated that the DASH score reflected their dietary pattern. In addition, 76% (22/29) felt that a DASH score of 10 (reflecting full adherence) was difficult to achieve. About one-third (10/29, 34%) reported that the text messages helped them reach their diet goals, and only 28% (8/29) felt the text messages were personalized.

**Table 3:** Perceived usefulness and ease of use of intervention components among participants receiving the DASH Cloud intervention (n=29).

Statements about DASH^a^ Cloud intervention components	Agreement with statement, n (%)
The feedback received on the automated text messages was helpful.	16 (55)
The DASH text messages helped me reach my personal diet goals.	10 (34)
The DASH text messages felt personalized.	8 (28)
The DASH text messages were sent at a convenient time each day.	21 (72)
I found the DASH score of 1-10 helpful.	16 (55)
I found the DASH score motivating.	13 (45)
The DASH score accurately reflected my diet pattern.	12 (41)
A DASH score of 10 was difficult to achieve.	22 (76)
I found the diet tips easy to understand.	23 (79)
I found the diet tips helpful to improve my DASH score.	16 (55)
I found the videos helpful.	19 (66)
I enjoyed watching the videos.	16 (55)
I learned a lot from the videos.	14 (48)
I applied the skills I learned from the video in my routine	14 (50)
Would you recommend this program to a friend or family member looking to eat healthy?	23 (79)

^a^DASH: Dietary Approaches to Stop Hypertension.

## Discussion

### Principal Findings

Adoption of the DASH dietary pattern can help with blood pressure reduction for the 100 million Americans with suboptimal blood pressure [3]. This study aimed to develop and test the feasibility of using a digital health tool that leverages smartphone diet-tracking apps to improve population-level adoption of DASH. We found that it was feasible to use a commercially available diet-tracking app in our intervention platform and add a behavioral intervention aimed at improving DASH adherence. We were successful at recruiting and retaining women with high blood pressure and achieved moderate to high rates of engagement with diet tracking across both study arms. Similarly, both study arms saw a small increase in DASH adherence at 3 months. However, we found that adding a digital behavioral DASH intervention to a diet-tracking app did not increase DASH adherence compared to diet tracking alone.

These findings are notable for various reasons. First, they signal that participants can achieve and sustain moderate to high engagement with diet-tracking apps even if no feedback is provided on app entries and that these apps can be used to improve their behavioral efficacy. Studies that have examined diet-tracking engagement without added behavioral intervention components demonstrated lower rates of tracking than the average of 4 days per week that we found in this study. In a study by Laing and colleagues [27], rates of diet tracking using the popular app My Fitness Pal dropped significantly after the first month. Patel and colleagues [28] found a similar steep decline in diet tracking after the first month. It may be that our focus on DASH rather than fitness or weight control motivated all participants regardless of whether they received feedback to engage more frequently.

Second, these findings also signal that any feedback is not always better than no feedback for improving engagement and overall dietary quality, with the caveat that we were not powered to evaluate those differences statistically. Behavior change theories and relevant empirical studies support that giving feedback, particularly tailored feedback, can improve engagement and subsequent behavior change [29,30]. However, the structure of the feedback, the level of personalization, and the relevancy of the feedback are important. Given the study design, we cannot know for sure why the intervention group tracked their diet less often as a result of the feedback; responses to the intervention satisfaction questionnaire give us some insight. Most notable is that less than half of participants found the DASH score used in the feedback motivating and reflective of what they ate. This finding does not preclude the use of “summary type” scoring feedback for improving dietary intake; rather, this suggests that we need to reconsider how to make it more helpful and interpretable to individuals. Additionally, as in any technology, we experienced bugs or issues with coding that did not line up with the logic outlined in the feedback algorithm. We aimed to test for these bugs before and during the execution of the intervention. However, it is possible that the presence of bugs could have impacted intervention engagement and perceptions about helpfulness of the feedback messages. We did not measure this in the study, so we are not able to quantitatively or qualitatively indicate how often this occurred or how much impact any bugs had on outcomes. Future studies should aim to consider measuring the impact of these bugs on outcomes.

Additionally, many participants stated that achieving full DASH adherence was difficult. Given this perception, the DASH score may not have provided positive reinforcement to support dietary behavior change. We are not aware of other studies that gave feedback on nutrients to improve diet quality, and we decided to use nutrient intake as part of the feedback because of its clear link to DASH adherence and the subsequent benefits on blood pressure. Participants may prefer feedback on adherence using food groups or some other measure of adherence. Overall, it would have been helpful to conduct a formal qualitative study after the intervention to better understand the discrepancies between the groups in engagement outcomes and determine how we could have improved satisfaction and perceptions of ease and helpfulness of the intervention. Future studies testing the feasibility of digital health interventions such as DASH Cloud should consider how best to understand the factors that will improve sustained engagement. We did not include a coach or any human support, and it may be that to change dietary behaviors, additional supports are needed beyond what can be provided via technology alone.

Third, these findings indicate that we may need to reconsider how to best provide personalized feedback to improve overall DASH adherence. Only 28% (8/29) of participants said they felt the texts were personalized, despite the use of an algorithm designed to personalize messages about intake of specific DASH nutrients. Our understanding of the most effective formula for personalizing feedback is mixed, but many studies support that personalizing interventions and feedback improves engagement [29,30]. We suggest that the personalization also reflect individual circumstances (eg, including feedback tips specific to one’s family structure) and attitudes and beliefs (eg, self-efficacy for changing behavior) and how those characteristics then impact the individual’s dietary behaviors. Future studies should work toward testing these different approaches when personalizing feedback. Despite the low ratings for the use of the DASH score and the personalization of the texts, 79% (23/29) of participants said they would recommend this program. This suggests that the overall concept of using diet-tracking apps to promote adherence to DASH is appealing.

The high rating of the program overall is important because of the substantial need to improve population-level adoption of DASH. Mellen and colleagues [15] used population-level data to assess DASH adherence shortly before and after the integration of DASH into national dietary guidelines. They found that less than 1% of the population is fully adherent to DASH and only 20% meet half of the DASH recommendations [15]. DASH adherence remained poor in subsequent cross-sectional population cohort studies [9]. As is supported by the current study, it may be that full DASH adherence is difficult to achieve. Indeed, trials testing intensive behavioral interventions to promote DASH have also struggled to help individuals achieve full adherence [31,32]. The PREMIER trial [33,34] was a comprehensive, multicomponent, behavioral intervention to improve adoption of DASH. The PREMIER intervention included standard behavior change components delivered via frequent face-to-face counseling with a registered dietitian and group meetings [33,34]. This high-intensity approach was effective in improving DASH adherence, but not full adoption of DASH [32]. This intervention also included other behavioral components, including sodium reduction, increased physical activity, and weight loss. Although comprehensive, focusing on multiple behaviors at a given time may have made it more difficult to fully adopt all DASH recommendations. Similarly, the ENCORE (Exercise and Nutritional Interventions for Cardiovascular Health) trial tested a comprehensive behavioral approach to adopting DASH, assessing the comparative efficacy of DASH to DASH plus behaviors for weight management on changes in blood pressure [35]. They found that both groups doubled their DASH adherence score, but the average score post intervention remained suboptimal [31]. Notably, the ENCORE trial found a linear relationship between DASH adherence and change in blood pressure. Full DASH adherence may be optimal, but partial adherence to DASH can be effective for lowering blood pressure [31].

The intensive intervention approaches tested in both PREMIER and ENCORE were effective in improving the adoption of DASH to clinically meaningful levels, but these approaches are not accessible to the broader population. To our knowledge, this was one of the first studies to test the feasibility of using mobile technologies and smartphone apps to disseminate and improve the adoption of DASH. Mann and colleagues [36] developed a similar intervention called DASH Mobile that consisted of easy tracking of DASH food portions; integrated Bluetooth blood pressure, weight, and pedometer monitoring; goal setting; simple data visualizations; and multimedia video clips to train patients in the basic concepts of the lifestyle change plan. This intervention has some notable distinctions from the current study. First, the intervention included more than just diet tracking and text message feedback and videos. It included other behavioral goals and behaviors to track, as well as the use of a coach to support behavior change efforts. Second, the research team developed the app rather than using commercially available ones to start. As such, it went through prototype testing and the researchers had to consider many elements of user design that for many commercially available apps have already been considered. Mann and colleagues [36] discuss lessons learned in the development of their DASH Mobile platform that are important for others looking to develop digital health programs to increase adoption of DASH. Our findings add to that evidence base but suggest that a lower-intensity approach improves DASH adoption only slightly. To truly extend the reach of evidence-based behavioral interventions to disseminate DASH, we need to continue to test ways to replicate what was done in PREMIER and ENCORE while maintaining the potential for dissemination we aimed for in DASH Cloud.

### Strengths and Limitations

Strengths of this study included the purposeful choice to use commercial apps in the intervention platform. This choice allows for more flexibility in future studies and increases the potential that this platform could be adapted for other technologies or outcomes. For example, if a new technology is developed that uses a different tool for tracking diet (eg, voice-assisted tracking), the intervention could be easily implemented with that new technology. This design choice increases the potential for dissemination. The use of a randomized controlled design allowed us to isolate the feasibility of the feedback and disentangle the effects of the tracking. Our results provide good insight about how feedback may not always improve engagement with diet tracking. Further, we achieved strong recruitment and retention rates, which supports the feasibility of this approach.

Despite these strengths, there are several limitations worth noting. This was a feasibility trial with a small sample size, so it is difficult to interpret the findings of this study because it was not powered to show an effect on changes in DASH adherence, engagement, or blood pressure. The primary focus was to assess the feasibility of developing the DASH Cloud platform by examining recruitment and retention rates and engagement with intervention activities, then exploring the effects of the intervention on DASH diet adherence and changes in blood pressure. As such, we did not conduct any power analyses. This is consistent with guidelines from Leon et al [21], which state that conducting power analyses is not needed when testing for feasibility. We also did not limit our study sample to only participants with hypertension or participants not taking hypertension medications. This was purposeful, given the focus on feasibility. However, this may have attenuated any differential effects on change in blood pressure. A common limitation of dietary studies is that the accuracy of the data collected is subject to potential recall and response biases [25,26]. Since participants were aware that their diet data would be collected, it may have led them to overestimate or underestimate dietary intake. Similarly, since we operationalized engagement using a valid day of tracking based on caloric intake, there may have been nondifferential misclassification of participants. Participants may have logged one meal or one food item to reach this threshold. Furthermore, the results of this study are not generalizable beyond the study population, which included mostly non-Hispanic White, educated women. The focus on recruiting only women was to better understand ways to reduce women’s disproportionate cardiovascular risk using lifestyle approaches to blood pressure management.

### Conclusion and Future Directions

We developed a digital program, DASH Cloud, that leverages commercially available diet-tracking apps that millions of Americans are using. In the current feasibility study, we found moderate to high engagement with diet tracking, an important predictor of successful behavior changes. However, this study also provided insights into the manner and type of feedback that should be given to participants using these diet-tracking apps. We need to better understand how to provide both personalized and relevant feedback to improve the uptake of DASH. To truly impact population health, we need to continually think not just about what best promotes the adoption of a healthy diet but also how to best disseminate and extend the reach of evidence-based treatments like the DASH dietary pattern.

## Abbreviations

API: application programming interface
ASA24: Automated Self-Administered 24-hour
CONSORT: Consolidated Standards of Reporting Trials
DASH: Dietary Approaches to Stop Hypertension
ENCORE: Exercise and Nutritional Interventions for Cardiovascular Health
NHLBI: National Heart, Lung, and Blood Institute
REDCap: Research Electronic Data Capture
USDA: United States Department of Agriculture
